# A Subject-Tailored Variability-Based Platform for Overcoming the Plateau Effect in Sports Training: A Narrative Review

**DOI:** 10.3390/ijerph19031722

**Published:** 2022-02-02

**Authors:** Ram Gelman, Marc Berg, Yaron Ilan

**Affiliations:** 1 Department of Medicine, Hebrew University-Hadassah Medical Center, Jerusalem 9103401, Israel; ram.gelman@gmail.com; 2 Department of Pediatrics, Lucile Packard Children’s Hospital, Stanford University, Palo Alto, CA 94304, USA; marc@area9.dk

**Keywords:** training, variability, muscle nerve tolerance

## Abstract

The plateau effect in training is a significant obstacle for professional athletes and average subjects. It evolves from both the muscle-nerve-axis-associated performance and various cardiorespiratory parameters. Compensatory adaptation mechanisms contribute to a lack of continuous improvement with most exercise regimens. Attempts to overcome this plateau in exercise have been only partially successful, and it remains a significant unmet need in both healthy subjects and those suffering from chronic neuromuscular, cardiopulmonary, and metabolic diseases. Variability patterns characterize many biological processes, from cellular to organ levels. The present review discusses the significant obstacles in overcoming the plateau in training and establishes a platform to implement subject-tailored variability patterns to prevent and overcome this plateau in muscle and cardiorespiratory performance.

## 1. Introduction

The plateau effect resulting from compensatory body adaptation mechanisms is described in exercise training and sports and applies to muscle and cardiovascular performance. The adaptation process is associated with several functional properties that occur over time during exercise. Muscle strength and fatigue adapt differently after several weeks of training to reach the improvement plateau.

Multiple mechanisms underlie the development of adaptation and plateaus during exercise, which presents a significant challenge in both healthy subjects and patients with chronic diseases.

This narrative review summarizes some of the mechanisms associated with the effect of plateaus in training and provides some recent examples from clinical trials. We describe some of the current measures used for overcoming plateaus in exercise. The review presents the concept of subject-tailored variability platforms for overcoming plateaus in training based on biological systems’ intrinsic variability. We describe the use of second-generation artificial intelligence systems which implement subject-tailored variability for improving the response to regular training.

## 2. Adaptation Mechanisms Are Associated with a Plateau in Muscle Performance during Exercise

Adaptation is a result of multiple mechanisms [1,2,3,4,5,6,7]. There is a change in the strength of the triceps brachii after training without correlation to fatigue rate (FR) or decrease in strength (DS). After four weeks of training, the FR and DS reach a plateau and do not change further with continued training [8]. Adding explosive and high-resistance interval training is associated with a significant sprint and endurance performance improvement, partly through improved exercise efficiency and anaerobic threshold. However, a plateau in the training effect appears after 8–12 sessions [9].

Neural adaptations in response to training occur at multiple levels of the neuroaxis. Three weeks of training are sufficient to induce significant compensatory adaptations in the neural drive to the muscles at the cortical level, attributed to alteration of the slow negative electroencephalographic activity termed as movement-related cortical potentials (MRCP) [10]. A recent study determined the role of exercise intensity and related central motor drive in defining corticomotoneuronal excitability in 10 subjects who performed a series of isometric single-leg knee extensions and bouts of cycling. Increases in exercise intensity and voluntary electromyogram (EMG) activity facilitated the corticomotoneuronal pathway similarly in isometric knee extension and locomotor exercise until a plateau occurred at submaximal exercise intensity. They reached the plateau at approximately 75% of the maximal capacity. Further increase in the contraction intensity did not lead to an additional change [11].

Strength training is associated with changes in the recruitment of motor units during voluntary contractions. A 3-week training period is associated with neural adaptations at the cortical level. Adaptations in the function of the motor unit evolve from changes in the synaptic input to the motor neuron pool or changes in the intrinsic properties of the motor neuron. The increase in muscle power after four weeks of training comes from the increased output from the motor neurons of the spinal cord to the muscles. After four weeks of strength training, adaptations in the tibialis anterior muscle motor units, such as increased motor unit discharge rate, decreased recruitment-threshold force of motor units, and an increase in motor neurons’ input-output, were noted. The normalized recruitment-threshold force of the motor units decreased after strength training, and there was an intensification in the discharge rate during the plateau phase of submaximal isometric contractions [12]. In a study comparing muscle activation while going to failure, fifteen healthy untrained women performed a set with heavy loading—at their three-repetition maximum (RM), and a set of repetitions to failure with lower resistance (approximately 15 RM) during dynamic shoulder abduction with elastic tubing. Throughout the failure set, normalized EMG was significantly lower during the first repetition and higher during the later repetitions, respectively, compared to the heavy loading set. Normalized EMG for the muscles increased throughout the set to failure and reached a plateau during the final 3–5 repetitions before the failure, suggesting that complete failure is not necessary to recruit the entire motor unit pool [13].

Several phenotypic characteristics are associated with the plateau effect. There are individual differences in response to training sessions, while the percentage of maximal strength determines the prescription. Subsequent sets are unlikely to induce more significant fatigue and activation of muscle after reaching a plateau in fatigue and repetitions per set [5]. Once a plateau is reached, heavy training does not further improve the performance of older runners with many years of training. On the other hand, heavy training can improve younger runners with fewer years of training [14].

In animal models, diffusion-weighted magnetic resonance imaging showed the progression of structural neuroplasticity induced by training. Rats underwent short training periods in the Morris water maze (MWM) task, which induced fast alterations in diffusion tensor imaging (DTI) indices. The earliest changes in DTI after 1 set of trials progressed with the second set but plateaued after the third [15].

These examples suggest that reaching a relatively early plateau during training is a significant challenge. 

## 3. Adaptation Mechanisms Are Associated with a Plateau in the Cardiovascular Parameters during Exercise

Adaptation to training is associated with a plateau effect in cardiovascular and respiratory parameters. The metabolic and respiratory events characterizing cardiopulmonary thresholds affect oxygenation in the brain and muscle, affecting the response of muscle recruitment [16]. Data from multiple studies in animal models suggested a close correlation between running speed and oxygen uptake, which changed to a steeper linear slope after strength training, thereby supporting an improved work economy with less oxygen consumption at fixed submaximal running speeds. The speed at which oxygen uptake reached a plateau increased in parallel with the change in maximal oxygen uptake during the training period [17].

In humans, changes in cardiorespiratory function plateau within several weeks of sprint interval training in a study comparing two sprint interval-training programs. The maximal change in peak VO_2_ occurred at three weeks of training. While endurance capacity, measured via time to exhaustion (TTE), showed maximal gain following nine weeks of training, a 50% reduction in the sprint duration did not decrease overall training adaptations [18]. In another study, twelve athletes repeated upper-body sprint exercises; the highest power was on the first sprint, with a progressive decline in power output over the next four sprints. The cycle rate remained unchanged while the work-per-cycle decreased progressively. The study shows a maximum decline in upper-body sprint performance with the initial five sprints, followed by highly de-saturated muscles and a plateau in pulmonary oxygen uptake after the initial 2–3 sprints. The maximum increase in VO_2_ occurred over the initial two sprints and plateaued at around 75% of the peak VO_2_ [19].

At the onset of exercise, oxygen transport adapts to meet the demands of the muscle. Cardiac output (Q) increases through the concomitant change in HR and stroke volume (SV), conditioned by the left ventricle (LV) function. SV increases concomitantly with LV strain and strain rates within the first 60 s of exercise, after which SV remains constant, and other systolic and diastolic parameters reach a plateau while LV untwisting continues to increase [20]. A study of 12 men suggested a plateau of VO_2_ after 8 h of ultra-endurance run. Subjects who maintained the highest maximal oxygen uptake during exercise experienced a higher deterioration of running (Cr) energy cost over 24 h on a motorized treadmill [21].

The effect of resistance training (RT) with different frequencies, including a follow-up period on cardiorespiratory fitness, was tested in healthy, older individuals. Improvements in oxygen consumption, cycling economy (CE), gross efficiency (GE), blood lactate concentration (La), and heart rate (HR) were observed during the initial three months, with no further improvements during the next six months of the study. Subjects maintained their maximal strength during follow-up, but CE returned to the baseline value [22].

A study of 25 cyclists who completed a maximal ramp test showed a non-linear increase in cerebral deoxyhemoglobin and oxyhemoglobin levels concomitant to the first ventilation threshold. Cerebral deoxyhemoglobin levels continued to increase while oxyhemoglobin levels reached a plateau or decreased after the second ventilation threshold. A threshold was identified for muscle parameters with a non-linear decrease in muscle oxyhemoglobin, attenuated muscle deoxyhemoglobin, and an increased EMG activity in the vastus lateralis (VL) and biceps femoris (BF) muscles. Thresholds in BF and VL EMG activity occurred after the second ventilation threshold [23].

The VO_2_ response to ramp incremental (RI) exercise does not always show a plateau-like effect at the tolerance limit. In a study among seven healthy male adults, RI cycle ergometry was performed to the limit of tolerance, followed by a 5 min recovery period and a step exercise (SE) test at 105% (RISE-105) of the final work rate (WRpeak) achieved during RI. Approaching the VO_2_peak did not reach a plateau during any RI or SE test component. However, a lack of change between the VO_2_peak established at different WRpeak values provides the criterion for a plateau to confirm maximum VO_2_ in a single test, even when the data response profiles do not suggest a plateau [24].

The VO_2_max is associated with a plateau in Q. Twelve young male cyclists performed incremental cycling and one-legged knee-extensor exercise (KEE) to exhaustion with and without right atrial pacing to increase their H.R. During control cycling, Q and leg blood flow increased by up to 85% of the maximal workload (WLmax) and did not change until exhaustion. SV increased initially, then plateaued and decreased before exhaustion, despite increased right atrial pressure (RAP). The study shows that atrial pacing increased the maximal heart rate (HRmax), but Q remained similar to the control condition at all intensities due to a lower SV No change in VO_2_. The data suggest that HRmax and myocardial work capacity do not limit VO_2_max. Limited filling of the left ventricle and altered contractility reduce SV during atrial pacing, whereas a plateau in LV filling pressure (LVFP) restricts Q close to VO_2_max [25].

Increased local blood supply underlies the oxidative adaptations to interval training regimens. Limb blood flow increases with the exercise intensity, but the muscle microvascular blood flow during incremental exercise reaches a submaximal plateau. During incremental exercise, perfusion and diffusion indices of microvascular oxygen transport to skeletal muscles plateaued at submaximal work rates. The study showed a similar effect even when limb blood flow and muscle recruitment increased [26]. The vascular strain is very high during heavy handgrip exercise. Occlusion and handgrip exercise for a long duration induces shear stress stimuli on the upper limit of the brachial artery (BA) and flow-mediated dilation (FMD). The stimulus created by brief occlusion and ischemic exercise is not increased by prolonging the occlusion or continuing to exercise post-occlusion [27].

The increased local blood supply mediates aerobic adaptations following interval training. In one study, 18 subjects performed 5 exercise bouts (30, 60, 90, 120, and 240 s) at 80% of peak oxygen uptake separated by 5 min recovery periods. Post-exercise oxygen availability and local blood supply increased significantly until the 90 s bout and plateaued thereafter [28]. Exercise intensities of ≥80% VO_2_peak induce the highest post-exercise oxygen availability locally. The results may be due to improved microvascular perfusion through lactate-mediated vasodilation [29]. However, there is no difference in mean response time (MRT) for blood flow or oxygen uptake with increased exercise intensity. When reaching maximal intensity, oxygen uptake appears to be regulated by blood flow and reaches a critical level at around 80% of WRmax. The data implies that high but not maximal exercise intensity may be an optimal stimulus for shear stress-induced small muscle mass training adaptations [30].

During ramp incremental cycling exercise, an increase in pulmonary oxygen uptake (VO_2_p) is matched by a linear increase in systemic Q. In a study of 20 male and female subjects, the change in near-infrared spectroscopy-derived muscle deoxygenation (Delta [HHb]) increased linearly with absolute VO_2_p during exercise and then reached a near-plateau, suggesting systemic blood flow, and thus oxygen delivery does not reflect the profile of blood flow changes at the level of the microvasculature [31].

A recent study determined the cardiovascular fitness and hemodynamic response to maximal cycle ergometer exercise tests in 425 children. The data showed a plateau or a decline in systolic blood pressure (SBP) close to the end of the test in one-third of the children, considered a part of the normal SBP response [32]. In another study, 64 stable congestive heart failure (CHF) patients performed a symptom-limited incremental exercise test, and a plateau or decrease in Q pattern was observed towards peak exercise in one-third of the patients, indicating a central hemodynamic exercise limitation [33].

Studies showed phenotypic differences for cardiovascular plateaus. The slow increase in the SV, SV index (SVI), and the cardiac index (CI), which reached a plateau, were observed in non-athletes while increasing progressively and continuously in athletes [34,35]. The type of exercise affects the plateau. Plateau incidence rates at VO_2_max differs between treadmill and cycle ergometry-based exercises [6]. In a study of 12-trained cyclists, priming exercise increased plateau response incidence at VO_2_max. The data showed a significant increase in the plateau response between the unprimed and heavy primed conditions during the final 60 s of the VO_2_max test [7]. One study evaluated markers of glycolytic metabolism in response to the Wingate and the incremental test in bike cyclists. Throughout the incremental test, the load was amplified every 3 min until exhaustion, and maximal aerobic power (APmax), VO_2_max, and time of VO_2_max plateau (Tplateau) were determined. Tplateau, VO_2_post differed between the mountain and road cyclists [3].

The gender-specific difference exists in the VO_2_max plateau associated with muscle oxygen extraction during incremental exercise. The study showed changes in deoxyhemoglobin (HHb) response during incremental exercise. Females have an earlier deoxyhemoglobin response (HHb) change with an earlier plateau [36]. Gender-based differences, such as the development of ventricular hypertrophy and an increase in VO_2_max, exist in response to 1 year of matched training. A progressive increase in males’ VO_2_max, LV mass, and mean wall thickness was described before reaching a plateau at 9–12 months of training. In contrast, there is a marked blunting of response among females despite the same training, with VO_2_max, LV mass, and mean wall thickness reaching a plateau after only three months of training [37].

Genetic difference in the VO_2_max plateau also exists. In a recent study, 26 male volunteers performed an exercise during which 14 of them reached the VO_2_max plateau. Subjects who achieved plateau were more likely to possess polymorphisms associated with anaerobic performance [38].

These examples suggest that a plateau in the cardiovascular parameters is a part of the body’s adaptation to training.

## 4. Adaptation Prevents Progress during Recovery in Patients with Neurological Diseases

The plateau effect is a significant obstacle to recovery from neurological events during exercise. A recent study determined the impact of high-intensity progressive resistance training (PRT) and high-intensity cycling in 48 chronic stroke patients. High-intensity P.R.T. improved peak power and muscle strength in both the affected and the unaffected lower limb. Strength improved during the initial 6–8 weeks and then plateaued over the final 2–4 weeks [39]. Functional electrical stimulation (FES)-supported interval training was associated with improved strength-endurance in the large muscle groups of the lower limb in motor-complete spinal cord injury (SCI). Ambulation capacity increased and then plateaued within a few training sessions in several patients [40]. High-volume electrically stimulated (ES) cycle training improved cardiorespiratory fitness and cycling power output in patients with paraplegia. The data showed reaching a training plateau by six months due to the physiology or the ES strategy used [41].

These data exemplify that adaptation is a significant obstacle during recovery in patients with neurological diseases.

## 5. Mechanisms Associated with the Exercise-Related Plateau

Several potential mechanisms mediate the exercise-associated plateau that regulates the body’s compensatory adaptation. Brain centers are associated with the regulation of these adaptation mechanisms. After several sessions of strength training, muscle strength starts increasing due to the alterations in the neural drive to the muscle as a result of adaptations at the cortical or the spinal level [12]. After a baseline training session, the primary and secondary motor regions show increased repetition suppression (RS). The data shows a decrease in motor system RS with continued training, while after the performance, it plateaus. Additional training leads to a pattern of increased RS in the contralateral sensorimotor cortex, the supplementary motor area, the ventral premotor cortex, and the anterior cerebellum, consistent with skill-specific specialization [42]. Low-intensity resistance exercise is associated with vasodilation of the external carotid artery (ECA) and vasoconstriction of the internal carotid artery (ICA). A study of 12 subjects that performed a one-legged static knee extension exercise showed that the ICA blood flow increased and reached a plateau before stabilizing 60 s into the exercise, despite a continuous increase in the cardiac output and the arterial blood pressure. The conductance of the ICA decreased at the end of the exercise [43].

The endocrine system plays a permissive and a regulatory role in the adequate response to exercise. High-intensity endurance exercise in professional athletes favors a catabolic response, depending mainly on the previous level of training. Athletes show significantly higher cortisol levels, except during the recovery period when the cortisol levels gradually decrease. Free testosterone decrease during the training period and a correlation between the sex hormone-binding globulin (SHBG) and the cortisol level is seen [44]. Another study examined the effect of high-intensity resistance and exercise on body composition and plasma leptin and ghrelin concentrations among 100 overweight subjects. The data showed increased exercise intensity accentuates body fat loss before a weight plateau. An initial decrease in body weight and fat continued at a lower rate for up to 3 months, stabilizing the rest of the protocol. Leptin levels decreased after day 21 and the third month and then stabilized. Ghrelin levels increased after day 21 and the third month and then returned to a level comparable to the baseline between 6–12 months when the body weight and fat had plateaued [45].

A study of a 30-km cycling trial showed that performance improves with training in the initial four weeks and does not change further in the last four weeks. Markers of oxidative stress (MDA concentration) and antioxidant status (CAT) in plasma during rest increased in response to training and may influence the performance plateau [46].

Protein synthesis in the muscles and muscle net balance plateaus following moderate protein ingestion. There is no limit to the anabolic response of the whole-body net balance to dietary protein. A recent study assessed whether whole-body muscle net balance plateaued in response to an increased protein intake during post-exercise recovery in adolescents and adults. The data showed an edge to the anabolic response to protein ingestion with a mixed meal. A higher intake leads to the deamination and oxidation of excessive amino acids. Adolescents support lean mass growth and have a higher anabolic sensitivity and capacity to assimilate dietary amino acids than adults [47].

The data shows that the exercise-associated plateau effect involves multiple mechanisms that make it challenging to overcome.

## 6. Maneuvers for Overcoming the Plateau Effect in Health and Disease

Manipulating the load component of the aerobic training session overcomes the muscle performance plateau in animals, which affects the physical performance of rats. Training protocols with a predominant overload in intensity (INT) or duration (DUR) or alternating and similar overloads in intensity and duration (ID) improved the physical performance of rats. The performance gain exhibited by the DUR rats reached a plateau after the fourth week. INT or ID rats did not reach a plateau and exhibited an increased performance at the end of the training protocol compared with the DUR rats. Compared with overload in the duration, overload in the intensity of training sessions was more effective at improving the performance over the eight weeks of the study [48].

In humans, the German Volume Training (GVT) method, involving ten sets of 10 repetitions, was traditionally used for increasing muscle mass. A recent study determined the effect of a modified GVT on muscular hypertrophy and strength among 19 males suggesting that this training program does not defer from performing five sets per exercise. 4–6 sets per exercise were optimal for maximizing hypertrophic training effects. Further, muscle gains plateau and may even regress due to overtraining [49].

A periodization is a practical approach to resistance training. A study determined the duration of performance increments, plateaus, and decrements among older women over nine weeks of isokinetic training. A plateau improvement was observed early during power (PWR) and strength (STR) training, suggesting that isokinetic training optimizing PWR and STR in older persons should use 3–4 weeks cycles. The average power curves of the PWR group showed a positive slope and then plateaued during the third week. The STR group peak torque (PT) curves for both legs showed initial positive slopes, which peaked between the 3rd and the fourth week and declined after that. The total work curves for both legs showed negative slopes across the first two weeks, steep positive slopes from the 3rd and the sixth weeks, and a final plateau [50].

In a study among 57 males, subjects’ physical fitness improved significantly during Finnish military 8-week basic training (BT) at the beginning of their service. A plateau in upgrading physical performance during a special training period (STP) evolved from a lack of continued progression or periodization in the training program [51].

Training using hypoxia at altitude improved physical endurance and overcame the personal plateau of the trained athletes [52]. Blood flow restricted resistance exercise (BFRRE) can increase muscle size and strength. BFRRE using low loads of 20–50% of 1 repetition maximum (1RM) is suggested for subjects who cannot tolerate high musculoskeletal forces and for trained athletes who reached a plateau in muscle mass and strength [53].

VO_2_max is essential for the assessment of middle- and long-distance running performance. Trained runners reach a plateau in VO_2_max, which can be enhanced using protocols that elicit 95–100% VO_2_max [54]. A study determined continuous graded exercise tests to exhaustion in 35 athletes. Continuous exercise limits the athlete’s ability to achieve a VO_2_max plateau. The use of a verification stage increases the frequency of achieving VO_2_max and can improve its measurement accuracy in athletes. After 10 min of active recovery, ten subjects (who did not demonstrate a plateau) completed a verification stage performed at supramaximal intensity [55].

Several measures are suggested for patients experiencing a plateau following a neurological event. Patients following chronic stroke often show a plateau or deceleration of motor recovery, leading to a discharge from physical therapy (PT). The motor function improves following the intensive practice of motor tasks. In patients discharged from PT because of a perceived plateau in motor function, intensive locomotor training (LT) after stroke improved daily stepping [56]. Robotic-assisted gait training (RAGT) improves gait function in rehabilitation after subacute or chronic stroke. In patients following a chronic hemiparetic stroke who reached a plateau after conventional training, RAGT combined with conventional PT improves the active torque, resistive torque, and stiffness in the paretic hip flexion phase [57]. In patients with hemiplegia after stroke, whose maximum walking speed reached a plateau, high-speed treadmill training improves the gait velocity. Training at a speed that is 20% faster than the maximum gait velocity of the day on the treadmill for five days increases the patient’s gait velocity [58]. In patients with subacute or chronic paraplegia or quadriplegia who reach a plateau in motor recovery after conventional therapy manifested by spasticity, RAGT improves the knee and hip kinematics and active knee joint movement while decreasing hip resistive force [59]. A study that used an intention-based robotic leg orthosis showed that lower extremity training improves gait speed following a chronic stroke after plateauing with exercise-based on a body-weight supported treadmill training program [60].

A study showed attenuation of the adaptations induced by twice-weekly resistance training sessions (RT) for 16 weeks in an older woman with sarcopenic obesity, improving adiposity indices, muscle strength, and functional capacity [61].

With small success in a limited number of subjects, most of these methods fail to address the exercise-mediated plateau effect.

## 7. Entropy, Variability, and Randomness Characterize Biological Systems: A Basis for Designing Methods for Overcoming Plateaus

Biological systems often do not behave according to pre-determined fixed rules. Their function changes over time and is unpredictable [62,63,64,65,66,67]. This dynamism is essential in both health and disease and is partly associated with the continuous need to respond and adapt to triggers to achieve new steady states [68,69,70,71,72,73,74,75].

Our incomplete understanding of the processes that underlie their function gives rise to this uncertainty. Entropy implies the information content and measures the rate of information gain and the degree of data irregularity. It quantifies the degree of regularity in a series and compares the complexity of different time series. A highly ordered and regular system has low entropy, while a state of complete randomness has maximum entropy [76,77]. Variability differs from randomness in biological systems, and distinctions between the two help avoid paradoxical results. Variability is the natural frequency of distribution associated with a variable, and it changes over time under steady-state conditions. It signifies the extent to which a distribution is stretched or squeezed as measured by the coefficient of variance (CV). Randomness is a specific type of incomplete knowledge that can be quantified, and it reflects different features of the system.

Spontaneous activity and firing among neurons communicate between neurons in the nerve-muscle axis. Nerves spike measurement suggests an equal probability of long or short spikes. The addition of a medium pattern of spikes increases the randomness as the number of choices increases due to the increased diversity. In contrast, the degree of variability decreases as the variation between the spikes is reduced [78].

A recent study determined the peak torque per repetition for subjects who performed repetitive concentric knee extension and flexion with an isokinetic dynamometer. The torque in the time series among healthy subjects or those with diabetes provided the non-linear features. A study in subjects with diabetes showed an increase in complexity and randomness in the variability of force produced by knee muscles. While the slope of flexor peak torques was lower in subjects with long-standing diabetes, they also had a lower fractal complexity. Subjects with diabetes for a short duration showed an increase in the randomness of force [79].

## 8. Variability in Biological Systems Characterizes the Typical Trajectory of the Body’s Response to Triggers: A Platform for Overcoming Plateaus

Variability is inherent to the normal function of body systems and can be identified at the genomic and the cellular levels or based on the function of whole organs [1,63]. It is described as the body’s response while attempting to reach an optimal steady-state or part of its compensatory adaptation to the continuous internal and external triggers. “Noise” in these systems is part of the process of identifying a steady-state and may affect both development and function [62,63,64,72,80]. The heterogeneity of the response to various triggers may be associated with a gradual accumulation of small amounts of this “intrinsic noise” [81]. At the genomic level, the RNA-MuTect technique helped identify somatic mutations using tissue-derived RNA samples and their matched normal DNA in healthy and tumor samples. In normal tissues, there are multiple somatic mutations. Genetic drift leads to some of these clones. A continuous response to multiple internal and environmental triggers may underlie this somatic mosaicism. Some mutations identified in the cancer genes may provide a selective advantage [82]. Loss of heart rate variability (HRV) or glucose variability is associated with a poor prognosis [83,84], which suggests that this variability is fundamental to the normal functioning of biological systems. HRV was used to monitor adaptation to exercise training [85]. Variability in response to drugs is beyond the pharmacogenomics and pharmacodynamics-based parameters, reflecting both inter-and intra-subjectively and between adjacent cells in the same tissue [86,87,88,89,90].

Some variability in movement is optimal for the normal function of muscle, as well as for therapy of neuromuscular disorders [91]. Muscle activity manifests a non-linear shape across the range of motions [91]. Variability characterizes regular human movements. The motor skills in a healthy state are associated with an optimal amount of movement variability characterized by chaotic patterns. In a study, 19 adults performed knee extension, knee flexion, and ankle plantarflexion isometric force contractions to target forces ranging up to 95% of maximal voluntary contraction at two angles. The force output was non-linear, and the joint angle affected the force-variability function for each muscle contraction [92].

Variability in muscle movement evolves from an “error” in predicting the motor program or an uncontrolled manifold [79]. It may be part of a systemic redundancy or “noise” that occurs due to multiple attempts in response to a given motor trigger. Similar to other biological systems, muscle variability is a usual attempt to cope with continuously changing internal and external triggers while maintaining a stable position during movement. An optimal state of variability, characterized by a chaotic structure, is required for a healthy and functional movement [93]. The variability in force during motion is associated with non-uniformity in the sarcomere length and depends on the joint angle, type of muscular contraction, muscle orientation related to the joint, and the synchrony and firing rate of motor units [79,92]. The non-linearity of muscle activity was associated with parameters that determine strength, including the speed of contraction, muscle length and composition, cross-sectional area, gender, and age [94,95].

Loss of the capability to express variability is associated with muscular rigidity or instability. Reduced variability is seen in Parkinson’s disease, while an increased variability may lead to instability [91]. An inverted U-shape relationship between the chaotic temporal variability during a steady-state is described [79]. A recent study evaluated the variability in angular limb displacement during a cross-country skiing gait. The data showed a chaotic behavior reflecting flexibility in the neuromuscular system to adapt to perturbations during skiing. At the state of fatigue, the chaotic behavior degraded, suggesting that the neuro-muscular system becomes unstable and less adaptable [96].

A study observed a pattern of muscular activity in the shoulder and arm during a repetitive dynamic task. During repetitive isokinetic exercise, with the onset of fatigue, there is a decrease in the overall limb output [97]. Isokinetic exercise is limited in the determined range and velocity, and the force produced is always in the chaotic and non-repetitive range [79,92]. The normalized mutual information between muscle pairs increases with time while the variability decreases. Variability in performance during the initial 10% of the task was unrelated to the time to task termination. The variability in force and posture masked the potential differences in the rate of development of fatigue among subjects [98]. Underlying conditions, including disease states, determine the non-linearity in the muscular force. The non-linear prediction analyzes muscle couplings, imbalance, and fatigue interactions during isokinetic exercise. The data showed that subjects with diabetes have muscular weakness and loss of its quality [99,100].

These data suggest that variability, complexity, and non-linearity characterize the function of muscles and necessitates the implementation of this variability for health and functional movement in therapies [67,74,101,102,103,104].

## 9. Overcoming Plateaus in Muscle Training by Introducing Subject-Tailored Variability Patterns

Overcoming plateaus in biological systems is a significant challenge to improve the response to chronic therapies [80]. Many currently used chronic medications or therapeutic maneuvers are associated with developing a plateau effect due to a partial or a complete loss of response over time with compensatory adaptations. Studies showed loss of response in over 40% of patients who received anti-TNF drugs [105,106]. Loss of effect is a significant challenge in around one-third of patients with epilepsy [107]. Drug resistance is a significant health issue during chronic opioid use for pain [108]. Studies showed a similar resistance for many other drugs like anti-depressants, anti-diabetics, and diuretics [64,65,75,80,102,103,104,109,110,111,112,113,114,115,116,117,118,119,120,121,122,123,124,125,126].

The body uses variability in many biological functions to optimize the response to the triggers. Introducing variability into therapeutic regimens is a method to overcome the plateau and resistance to chronic therapies. Introducing simple regimens of alternating dosage or drug holidays can help improve the response to chronic therapies. Intermittent dosing of rapamycin, with drug holidays, improved the anti-epileptogenic properties in mouse models of tuberous sclerosis complex (TSC) [127]. In a prospective trial on patients with inflammatory bowel disease, those who underwent an intervention with an increase or decrease in the dose of anti-TNF maintenance treatment had a 13% reduction in the loss of effect compared to 36% in those who received fixed-dose regimens [128]. A study showed dose reductions in non-cancer patients with pain on chronic opioid therapy who are at a higher risk of opioid-related adverse outcomes [129]. A review of 67 studies aimed at reducing opioid dose suggested that several interventions effectively reduce or discontinue chronic opioid use while alleviating pain and improving quality of life [81,130].

Second-generation artificial intelligence systems can implement variability into training and therapeutic regimens for overcoming biological tolerance and plateaus for various chronic diseases [80,102,103,104,112,113,114,115,116,117,118,119,120,121,122,123,124,125,126].

**Figure 1:**
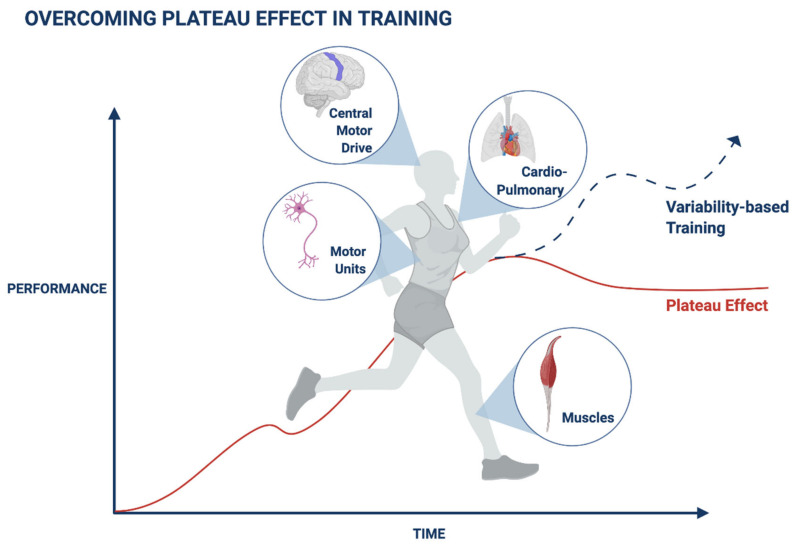
A schematic presentation of a method for overcoming plateaus and improving performance by introducing variability into training programs.

Figure 1 shows a schematic presentation of improving performance by introducing variability into training.

Introducing variability to improve muscle performance can be implemented in two stages. In the first stage, variability in training involves alternating the magnitude of strength and additional parameters of muscle stimulation patterns. In the second stage, individualized variability patterns, whether directly or indirectly related to muscle performance, can be integrated into the variability algorithm for improving performance output [80,113,126]. Regular, fixed-based muscle training regimens may be incompatible with the variability that characterizes the muscle–nerve–brain axis, thereby contributing to the plateau effect, which precludes continuously improving performance [126]. Variability signatures can be quantified and implemented to improve the algorithm’s performance in health and disease states [80,113,126,131].

## 10. Conclusions

To summarize, biological systems are self-organized under the environmental, biochemical, and morphological constraints to obtain the most balanced state [79]. Compensatory adaptation mechanisms characterize the muscle–nerve–brain axis while responding to triggers contributing to the plateau effect during muscle training. The body uses variability patterns to optimize the function of various systems. Ongoing studies examine the ability to provide variability-based regimens tailored for subjects to improve muscle performance continuously.

## Abbreviations

MRCP: movement-related cortical potentials; EMG: electromyographic activity; RM: repetition maximum; FR: fatigue rate; DS: decrease in strength; HHb: deoxyhemoglobin; MWM: Morris water maze; DTI: diffusion tensor imaging; RT: resistance training; CE: cycling economy; GE: gross efficiency; La: lactate; HR: heart rate; O2: oxygen; SV: stroke volume; LV: left ventricle; RMT: respiratory muscle training; Cr: cost of running; 24TR: 24 h; treadmill; VO_2_max: maximal oxygen uptake; VL: vastus lateralis; BF: biceps femoris; RI: ramp incremental; SE: step exercise; WR: work rate; Q: cardiac output; KEE: knee-extensor exercise; WLmax: maximal workload; RAP: right atrial pressure; HRmax: maximal heart rate; LVFP: LV feeling pressure; BA: brachial artery; FMD: flow-mediated dilation; MRT: mean response time; VO_2_p: pulmonary O2 uptake; Delta [HHb]: delta in muscle deoxygenation; APmax: maximal aerobic power; Tplateau: time of VO_2_max plateau; SVI: SV index; CI: cardiac index; SBP: systolic blood pressure; CHF: congestive heart failure; PRT: progressive resistance training; FES: functional electrical stimulation; SCI: spinal cord injury; ES: electrically stimulated; RS: repetition suppression; RAGT: Robotic-assisted gait training; HRV: heart rate variability.
